# A Noble Work

**Published:** 1907-09

**Authors:** 


A Noble Work.—Dr. Margaret R. Holliday, late assistant physician to the State Lunatic Asylum at Austin, and Mrs. Dora Mc-

Daniel, late matron to the same institution, have established in Austin a school, home, and sanatorium for the care of defective children. There is no institution of the kind provided by the State, and none in Texas. Such an institution is greatly needed, and I am sure will be appreciated. The movement and the management have the support and endorsement of the medical staff of our insane asylums and the profession of Austin. Dr. Holliday, the superintendent, is a highly cultivated lady, who has had wide experience in this field. She took the degrees of B. S. and M. S. at the University of Texas and M. D. at the Medical Department of the University. In her prospectus, a copy of which can be had free on request, she says:

"Children suffering from mental and physical defects, due to prenatal influences or to injury and disease in infancy and early childhood, which has caused arrested development mentally and anatomical defects physically, are the ones to be aided. These children are a great care in the home, where it is impossible to give proper attention to their physical needs or mental training, both from lack of necessary time and skill. Those seeking a retreat for the neurotic child, which should have a special training and care during the growing period to prevent a mental breakdown when the stress of adolescence and adult life place an additional burden on a nervous organization too weak to stand the strain, will here find the place for which they are seeking. The hysterical child, who should be taken away from home influences and sympathy, will receive special attention and medical care in the effort to return it to a normal condition. Children suffering from chorea, partial paralysis and other nervous affections, which render normal training impossible, but who should not be neglected, will receive constant medical attention and given mental training, controlled by the individual needs and ability of the child. The child suffering from physical defects, as hair-lip, disfiguring burns, wasted limbs, and so on, who are morbid as regards their condition and, therefore, shun the gaze and remarks of the thoughtless, will find here a home where their defects are lost sight of and their morbidity dispelled. The child backward at school, as well as the child disagreeable and unmanageable in the home, will be under close scrutiny to detect any abnormalities never suspected as adenoids, defective vision, nasal troubles, which will be corrected and an attempt made to render the child capable of taking his place by the side of normal brothers and sisters. The child with arrested mental development can not be transformed into a normal child, but can be taught correct habits, to care for themselves, and thus rendered less a burden than when neglected and ignored, and their unfortunate condition demands that they be given proper attention and training.

"In addition to these features the sanitarium will be prepared to care for and treat purely nervous cases in adults, where absolute quiet, constant treatment and hospital life is demanded."

